# *Strongyloides stercoralis* infection in dogs in Austria: two case reports

**DOI:** 10.1186/s13071-022-05270-2

**Published:** 2022-05-15

**Authors:** Maria Sophia Unterköfler, Iris Eipeldauer, Sophie Merz, Nikola Pantchev, Josef Hermann, René Brunthaler, Walter Basso, Barbara Hinney

**Affiliations:** 1 Institute of Parasitology, University of Veterinary Medicine Vienna, Vienna, Austria; 2 IDEXX Laboratories, Kornwestheim, Germany; 3 Veterinary Practice Dipl.Tzt. Josef Hermann, Trautmannsdorf, Austria; 4 Institute of Pathology, University of Veterinary Medicine Vienna, Vienna, Austria; 5 Institute of Parasitology, Vetsuisse Faculty, University of Bern, Bern, Switzerland

**Keywords:** Zoonosis, Baermann funnel technique, *COI*, *18S* rDNA HVR I, *18S* rDNA HVR IV, *Strongyloides*, Moxidectin, Ivermectin

## Abstract

**Background:**

*Strongyloides stercoralis* is endemic in tropical and subtropical regions, but reports of infections in central and northern Europe have been recently increasing. Infections occur mainly in humans and dogs. In dogs, both dog-adapted and zoonotic *S. stercoralis* genotypes seem to occur. Clinical manifestations mainly include gastrointestinal and respiratory signs. The severity of the disease can vary greatly and depends on the immune status of the host. The infection is potentially fatal in immunosuppressed individuals, either medically induced or due to an underlying disease, in which hyperinfections and disseminated infections with extraintestinal parasite dissemination may occur.

**Methods:**

Diagnosis was based on coproscopy, including flotation and the Baermann funnel technique, histology of small intestinal biopsies and molecular analysis of mitochondrial cytochrome oxidase subunit I (*COI*) and hypervariable regions I and IV (HVR I and HVR IV) of the nuclear* 18S* rDNA loci.

**Results:**

Two independent cases of severe canine *S.* *stercoralis* infection in Austria are presented. In both cases, *S. stercoralis* was detected in histological sections of the small intestine and with the Baermann funnel technique. Molecular analysis revealed strains with zoonotic potential. Case 1 was a 1-year-old female French bulldog with a long history of respiratory and gastrointestinal signs, severe emaciation and apathy before *S.* *stercoralis* infection was diagnosed. Treatment with moxidectin (2.5 mg/kg body weight [BW], oral route) did not eliminate the infection, but treatment with ivermectin (0.2 mg/kg BW, subcutaneously) was successful. Case 2 consisted of two 2-month-old Pomeranian puppies, one female and one male, from a litter of four, which died soon after presenting dyspnoea and haemorrhagic diarrhoea (female) or torticollis (male); *S.* *stercoralis* infection was first diagnosed post-mortem.

**Conclusion:**

More attention should be paid to this nematode because although it appears to be rare in Austria, it is easily overlooked on standard coproscopy unless a Baermann funnel technique is used, and even then, it can be missed. Moxidectin is not always successful in eliminating the infection, and treatment with ivermectin should be considered in cases of infection.

**Graphical Abstract:**

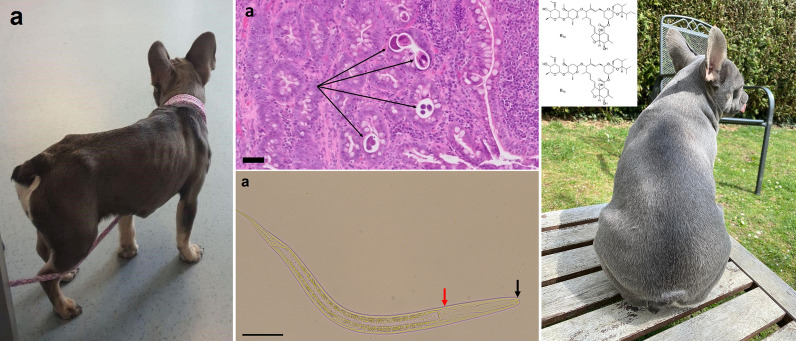

## Background

*Strongyloides stercoralis* is an important soil-transmitted helminth in humans and dogs living in tropical and subtropical endemic areas, with the highest prevalence reported in Southeast Asia, sub-Saharan Africa and Latin America [1–3]. Europe is considered to be a low-endemic area, with most autochthonous human cases having been reported in Spain, Italy and France [4] although human cases have been reported from several other European countries [4–6]. There have been case reports of *S. stercoralis* infection in dogs from several European countries, with the most coming from Italy [4, 7–11]. In Austria, the most recent case report in dogs was published in 1985 [12]. More recently, however, canine *S.* *stercoralis* infection was reported from Switzerland in 2019 [13] and 2022 [14] and from the UK in 2020 [15].

*Strongyloides stercoralis* is a small and slender nematode belonging to the order Rhabditida. Its life-cycle is complex. Only parasitic females reside within the small intestine, where they produce embryonated eggs through mitotic parthenogenesis. In the environment, two different pathways (homogonic or heterogonic cycles) are possible. First-stage larvae (L1) are excreted with the faeces and either develop to infective third-stage larvae (L3) in the environment (homogonic cycle), or into male and female adults, which reproduce to generate offspring, which will then develop into infective L3 (heterogonic cycle). The infection occurs mainly percutaneously or through oral uptake of L3 (from the environment or through transmammary transmission), followed by tracheal or somatic migration of the larvae. Autoinfection has also been described, and it may result in very high infection levels. *Strongyloides stercoralis* can infect dogs, wild canids, humans, non-human primates and cats [16, 17]. Two genetically distinct populations of *S.* *stercoralis* were found in dogs from Cambodia, with one strain found only in dogs and the other strain shared with humans in that region [18]. Differentiation of potentially zoonotic strains from non-zoonotic strains is usually based on analysis of the mitochondrial cytochrome oxidase subunit I gene (*COI*) and hypervariable regions I and IV (HVR I and HVR IV) of the nuclear* 18S* rDNA loci [13, 18–21].

In dogs, infections with *S.* *stercoralis* can be asymptomatic. However, mainly in young animals with a high worm burden, gastrointestinal and respiratory signs, as well as skin lesions, might occur. Especially in immunodeficient individuals, hyperinfection with the dissemination of the parasite to visceral organs can lead to more severe clinical signs and even death [16, 22]. Severe hyperinfection may also be induced by glucocorticoid administration [13]. Diagnosis of *S. stercoralis* is usually based on coproscopy with the Baermann funnel technique, while the flotation technique does not reliably detect the L1. However, it may be useful to detect eggs or adult females, which also may be occasionally excreted [13]. Other diagnostic techniques include the agar plate culture and serological and molecular tests [23–25]. No controlled clinical trials have been performed to identify suitable drugs for the treatment of *S. stercoralis* in dogs, although several case reports have shown that treatment with ivermectin, as well as with fenbendazole or moxidectin, often in combination, were able to treat the infection. However, single-dose application in these case reports did not reliably eliminate the parasite, and repeated follow-up examinations and treatments were often necessary [13, 26, 27].

Here, we report the diagnosis, including molecular strain identification, treatment, follow-up and outcome of two recent cases of canine *S.* *stercoralis* infection in Austria.

## Methods

### Coproscopy

For flotation, faeces were suspended in glucose-sodium chloride solution (density: 1.33 g/ml) and centrifuged at 690 *g* for 4 min. For the Baermann funnel technique, faeces were wrapped in gauze and placed in a sieve and funnel. The funnel was filled with water and migrated larvae were collected after 6 to 12 h.

### Histology

Histological sections of small intestinal biopsies were embedded in paraffin using standard methodology, stained with hematoxylin and eosin and evaluated by light microscopy.

### Molecular analysis

Molecular analysis was performed to confirm the diagnosis and obtain information on the genetic background and zoonotic potential of the *S.* *stercoralis* specimens. DNA was extracted from L1 obtained by the Baermann funnel technique (case 1 and from the pooled sample of both puppies in case 2) using a commercial DNA extraction kit (DNeasy® Blood & Tissue Kit; QIAGEN, Hilden, Germany) according to the manufacturer’s instructions, and from formalin-fixed paraffin-embedded (FFPE) intestinal tissue (i.e. intestinal biopsy of case 1), as previously described [28]. The following PCR primer pairs were used: COIintF/COIintR, targeting the mitochondrial *COI* gene [29]; SSUA/SSU26R, targeting the HVR I of the nuclear* 18S* rDNA [30]; and 18SP4F/18SPCR, targeting the HVR IV of the nuclear* 18S* rDNA [19]. The PCR protocols used were described in detail in an earlier report [13].

## Results

### Case 1

Case 1 was a 1-year-old female French bulldog, originating from Slovakia, rescued from an animal shelter and living in a household in Austria, southern Styria with two other dogs and three cats. The dog was vaccinated against canine distemper virus (CDV), canine adenovirus (CAV) and canine parvovirus (CPV-2) at the age of 2 months, followed, 3 weeks later, by vaccinations with the second dose of CDV, CAV, and CPV-2 together with vaccinations against canine parainfluenza virus (CPiV) and *Leptospira* spp. After a further 4 weeks, the dog received the third dose of vaccination against CDV, CAV, CPV-2 and the second dose against CPiV and *Leptospira* spp., as well as vaccination against rabies (Nobivac®; Intervet GmbH, Unterschleißheim, Germany). At the age of 7 months, the dog was brought to Austria and at that time point it was dewormed with a spot-on combination compound (Moxiclear®; Norbrook Laboratories Ltd., Monaghan, Ireland) containing moxidectin (2.5–6.25 mg/kg body weight [BW]) and imidacloprid (10–25 mg/kg BW). Coughing, non-response to antibiotics and intermittent diarrhoea were reported at that time. Respiratory signs resolved spontaneously within 2 months, and a diagnosis of *Giardia* infection was made using a rapid immunoassay test (SNAP® *Giardia* Test; IDEXX, Vienna, Austria), but gastrointestinal signs, including vomiting and wasting, continued despite treatment with fenbendazole (50 mg/kg BW, oral route [p.o.], one dose daily [SID], 5 days; Panacur®; Intervet GmbH, Vienna, Austria). After 3 months, the dog developed a haematoma and emphysema on the head following an accident, which were treated with a single dose of dexamethasone (0.5 mg/kg BW, subcutaneously [s.c.]; Dexa “Vana”; VANA GmbH, Vienna, Austria).

**Fig. 1:**
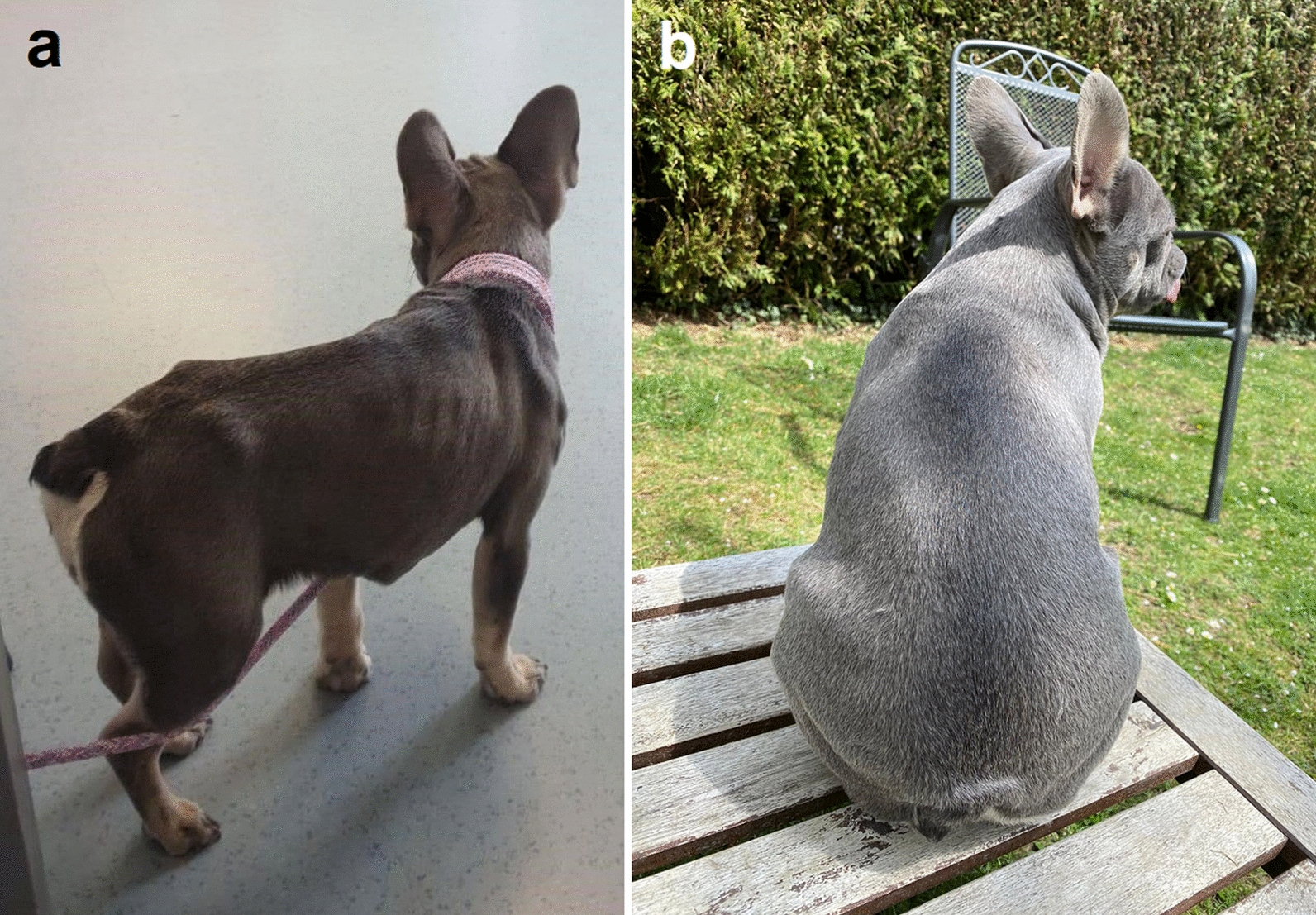
Photographs of case 1 before diagnosis of *Strongyloides* *stercoralis* infection (**a**) and after treatment with ivermectin (**b**). Notice the clearly visible bony structures of the ribs, shoulders and hips, indicating poor body condition and the dull hair coat in **a** as compared to the good body condition with fat covering the bony structures and shiny hair coat in **b**

With worsening of the gastrointestinal signs, a malabsorption syndrome was suspected. A diet change from standard to dietary feed (Hill’s PRESCRIPTION DIET® a/d; Hill’s Pet Nutrition GmbH, Hamburg, Germany) was made, but no improvement was noticed. Without previous coproscopical examination, the dog was treated with a combination product p.o. (Milbemax®; Elanco GmbH, Cuxhaven, Germany) containing milbemycin (0.5 mg/kg BW) and praziquantel (5 mg/kg BW). After 1 month, the dog still had a poor general condition and was apathetic (Fig. 1a). An ultrasonographic examination showed increased wall thickness of the small intestine (5.4 mm), leading to the suspicion of inflammatory bowel disease (IBD). Treatment with prednisolone (1 mg/kg BW, p.o., SID; Prednicortone®; Le Vet Beheer B.V., Oudewater, The Netherlands) was initiated, and the dog received another dose of milbemycin (0.5 mg/kg BW) and praziquantel (0.5 mg/kg BW) p.o. (Milbactor®; KRKA d.d., Novo mesto, Slovenia).

After an initial improvement of the clinical signs within 2 weeks, the dog developed acute diarrhoea and was treated with prifinium bromide (0.075 mg/kg BW, s.c.; Prifinial®, Vetoquinol GmbH, Vienna, Austria) and sulfasalazine (25 mg/kg BW, p.o., twice daily [BID]; Salazopyrin®; Pfizer Corporation GmbH, Vienna, Austria). Because the clinical signs did not improve, after 2 days another SNAP *Giardia* test was performed, which was again positive. The dog was treated with metronidazole (50 mg/kg BW, p.o., BID, 5 days; Metrobactin®, Le Vet Beheer B.V.). As the condition of the dog did not improve within the next month, metronidazole and sulfasalazine were repeatedly applied in the same dose and formulation.

**Fig. 2:**
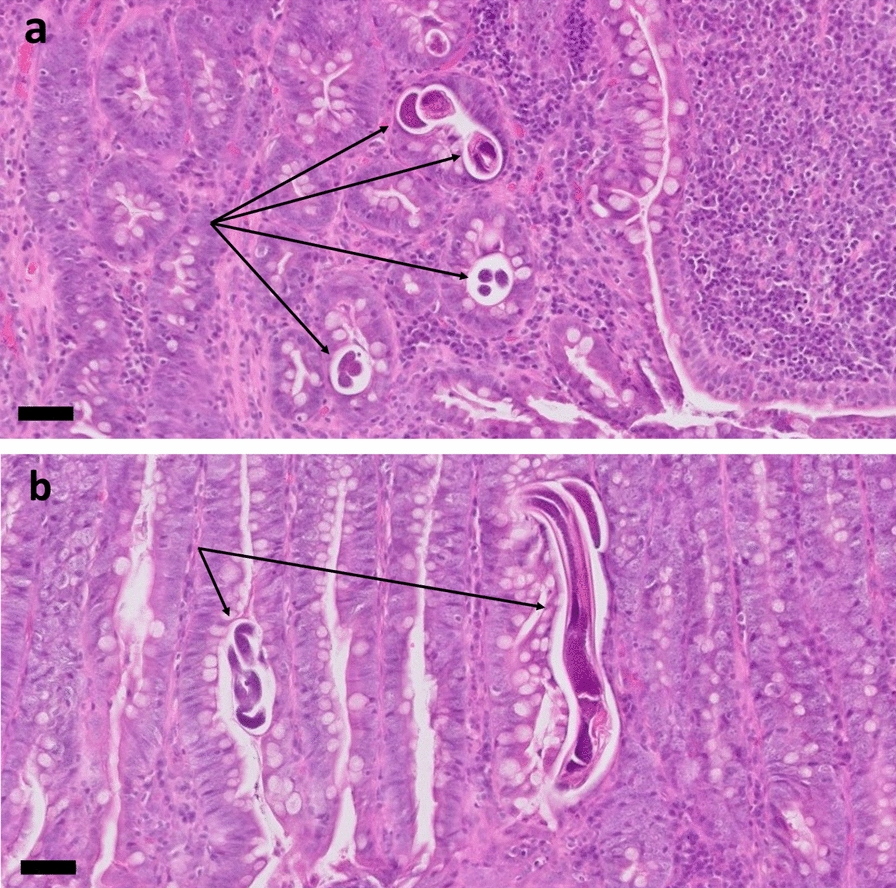
*Strongyloides* (arrows) in histological section of the small intestine from case 1 stained with hematoxylin and eosin (H&E) showing cross-sections (**a**) and longitudinal sections (**b**). Images taken at ×200 magnification. Bar: 50 µm

Two weeks later the gastrointestinal signs worsened, and a full-thickness biopsy of the small intestine was performed under general anaesthesia. Histologic examination was performed at the IDEXX Laboratories, and a moderate, chronic, lymphoplasmacellular and eosinophilic enteritis with multifocal crypt abscesses and numerous nematode stages in the mucosal layer was observed. Nematodes within the intestinal wall were diagnosed as *S.* *stercoralis* stages (Fig. 2). Within the histology sections of the gut, a paired genital tract, a small size of the parasite and a relatively large intestine are features of *Strongyloides* [31].

**Fig. 3:**
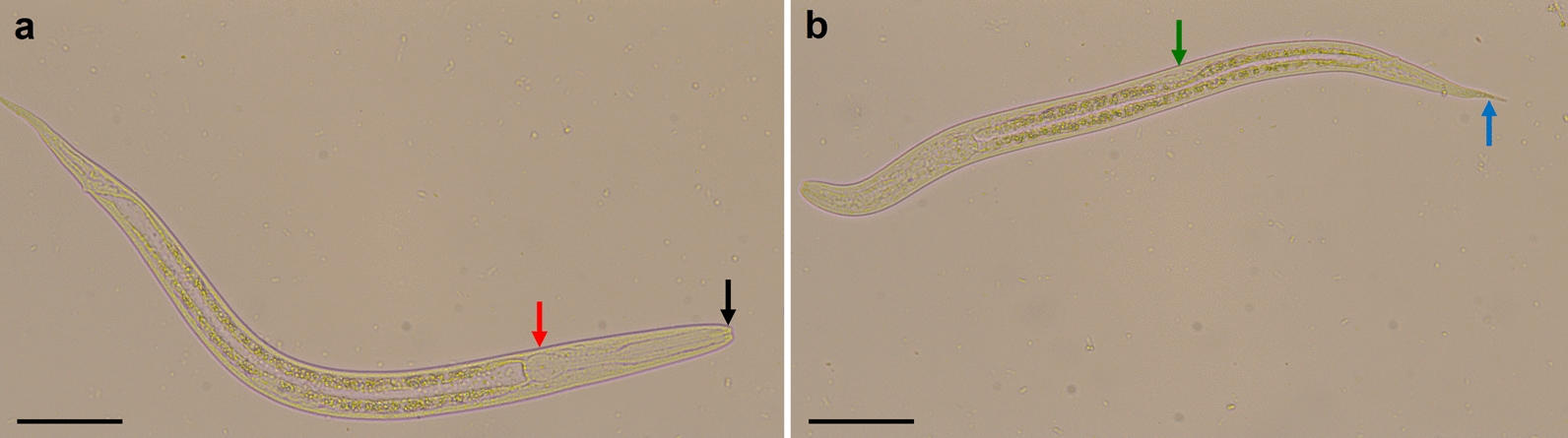
Unstained light microscopic image of larvae isolated with the Baermann funnel technique from case 1. Small buccal capsule (**a**, black arrow), rhabditoid oesophagus (**a**, red arrow), prominent genital primordium (**b**, green arrow) and straight tail (**b**, blue arrow) are characteristic for first-stage larvae of *S.* *stercoralis*. Images were taken at ×400 magnification. Bar: 50 µm

This result was confirmed by coproscopy with the Baermann funnel technique (Fig. 3). The two other dogs, three cats and two humans living in the same household did not show any clinical signs of infection and were examined for *S.* *stercoralis* infection by means of flotation and the Baermann funnel technique. In one of these two other dogs in the same household, *S.* *stercoralis* larvae were detected by the Baermann funnel technique and *Toxascaris leonina* eggs by the flotation method. The remaining dog and the three cats tested negative by both techniques at that time point. The owners were instructed to follow personal and environmental hygiene measures, including cleaning the floors and surfaces with a steam vacuum cleaner, machine washing textile fabrics at a minimum temperature of 60 °C and cleaning the animals’ feeding and water bowls at a minimum temperature of 60 °C in the dishwasher. Prednisolone therapy was tapered over 2 weeks and subsequently stopped. The patient dog, as well as the two other dogs and three cats living in the same household, received six times a combination compound containing moxidectin (2.5 mg/kg BW) and imidacloprid (10 mg/kg BW) spot-on every 4 weeks (Advocate®; Bayer AG, Leverkusen, Germany) and three times at the same time point a combination compound containing milbemycin and praziquantel, as described above. The other dog that was affected was negative for *S.* *stercoralis* larvae in coproscopy after 1 week.

The patient dog’s condition improved clinically, but the faeces were still positive for *S.* *stercoralis* larvae by the Baermann funnel technique at 1 and 2 weeks after the first treatment with Advocate^®^, and *T.* *leonina* eggs were detected in the second faecal examination by flotation. The dog was additionally treated with sarolaner (2.4 mg/kg BW), moxidectin (0.048 mg/kg BW) and pyrantel (10 mg/kg BW) p.o. (Simparica trio®; Zoetis, Louvain-la-Neuve, Belgium). Because the dog continued vomiting, a lateral abdominal radiograph was performed, which showed increased opacity at the pylorus region. Gastroscopy was performed under sedation, and a biopsy of the thickened and calcified pylorus region was performed. No foreign body could be detected, and histologic examination of the biopsy revealed no abnormal results.

At 2 and 6 weeks after anthelminthic treatment with Simparica®, *S.* *stercoralis* larvae could still be detected with the Baermann funnel technique. The SNAP *Giardia* test and a rapid immunoassay test for *Cryptosporidium* (FASTest® CRYPTO Strip; MEGACOR, Hörbranz, Austria) were negative. Analysis of the multidrug-resistance (*MDR-1*) gene (TransMIT, Gießen, Germany) showed no defect, and the dog was treated off-label with ivermectin (0.2 mg/kg BW, s.c.; Ivomec®; Boehringer Ingelheim, Lyon, France). No larvae could be detected with the Baermann funnel technique after 2 and 4 weeks. The second of both other dogs living in the same household tested positive for *S.* *stercoralis* at this time point and subsequently treated two times with ivermectin in the described dose and formulation within a 2-week interval after testing negative for the *MDR-1* gene defect. In the patient dog, three months after treatment with ivermectin one dead and degenerated larva was detected with the Baermann funnel technique; at 4 and 5 months after ivermectin treatment, no larvae were detected. The patient was clinically normal and showed normal weight (Fig. 1b). The two dogs living in the same household as the patient dog were examined by means of flotation and the Baermann funnel technique every time the patient dog was examined, and no larvae could be detected except at the mentioned time points.

### Case 2

**Fig. 4:**
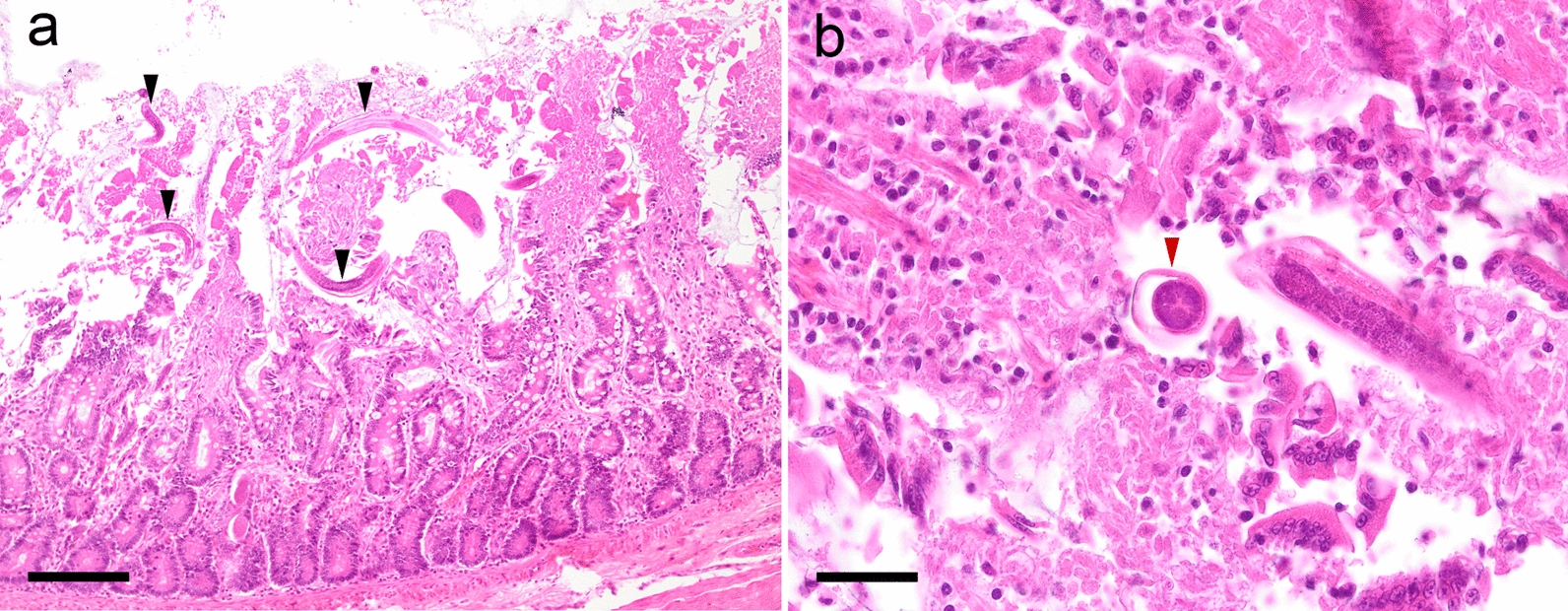
Histological section of the small intestine from case 2 stained with H&E. Longitudinal sections of larvae (black arrowheads) in the section of the female puppy (**a**) and cross-section (red arrowhead) of a larva in the section of the male puppy (**b**). Images were taken at: **a** ×100 magnification, bar: 160 µm; **b** ×400 magnification, bar: 40 µm

Case 2 involved two Pomeranian puppies, aged 2 months, from a breeder in Austria, southern Styria, who owned 23 Pomeranian dogs at ages ranging from 1 to 7 years. Adult dogs were regularly dewormed with a spot-on combination compound containing moxidectin (2.5 mg/kg BW) and imidacloprid (10 mg/kg BW) every 2 months (Advocate^®^; Bayer AG), and puppies were dewormed at the age of 3 and 6 weeks with fenbendazole (50 mg/kg BW, p.o., SID, 3–5 days; Panacur®; Intervet GmbH, Vienna, Austria). After that, they were routinely dewormed together with the adult dogs at the time points mentioned above. Several months before the puppies were born, the breeder had imported two dogs from Russia, and from that time point onwards, clinical respiratory and gastrointestinal signs began to appear intermittently in the population. The litter consisted of one female and three male puppies. The female puppy showed dyspnoea and mucous-haemorrhagic diarrhoea and was treated with antibiotics but died after treatment. One of the male puppies showed torticollis, but no gastrointestinal signs and died suddenly before any treatment could be initiated. The other two male puppies did not show clinical signs and no further examinations were performed. The dead puppies were sent in for pathological examination. Macroscopic and histologic examinations of the lungs showed no pathological changes. In histological sections of the small intestine from both puppies, larval worm stages were found in the mucosa (Fig. 4).

A pooled faecal sample from both puppies was sent post-mortem for a parasitological examination. *Cystoisospora ohioensis* oocysts and *S. stercoralis* larvae were detected by the flotation and Baermann funnel techniques, respectively, and a positive SNAP *Giardia* test (see above) was observed. Liver, lung and spleen samples from both puppies were pooled and analysed as one sample by PCR for CDV, CAV1, CAV2, CPiV, canine herpesvirus (CHV-1), canine respiratory coronavirus (CRCoV), influenzavirus A, and canine bocavirus (CBoV) at the Institute of Virology, University of Veterinary Medicine Vienna. All PCR results were negative except for CBoV.

All remaining dogs in the population were treated twice with ivermectin (0.5 mg/kg BW, p.o.; Ivomec®, Boehringer Ingelheim, Lyon, France) at an interval of 2 months without previous coproscopical examination. Three months later, faecal samples from 24 dogs present at the breeding unit at that time point were examined by coproscopy. *Strongyloides stercoralis* could not be detected with the Baermann funnel technique. Additional findings were *Cystoisospora canis* oocysts in two dogs and *Uncinaria stenocephala* eggs in two further dogs by flotation, as well as a positive SNAP *Giardia* test for two further dogs.

### Molecular analysis

In case 1, all three DNA markers could be successfully amplified with L1-derived DNA, and *COI* and* 18S* rDNA-HVR I loci could also be amplified from FFPE-intestinal biopsy-derived DNA. There were no differences in the amplified sequences obtained from both DNA samples. In case 2, only the* 18S* rDNA-HVR I marker could be amplified. All amplicons showed 99–100% sequence identity with *S. stercoralis* GenBank entries, confirming the morphological diagnosis.

In case 1, the amplified *COI* sequence showed 100% (649/649 bp) identity with *S. stercoralis* sequences from three dogs from Switzerland (accession nos. MH932101-MH932103) and one dog from USA (accession no. AJ558163). The amplified* 18S* rDNA-HVR I sequences of parasites in case 1 and case 2 were identical. These sequences had 99.9% (820/821 bp) identity with *S. stercoralis* sequences isolated from dogs in Switzerland (accession no. MH932098 and MH932099) and corresponded to the haplotype VI of HVR I. This haplotype has been previously observed in *S. stercoralis* infecting dogs and primates [13].

In addition, parasites isolated from case 1 displayed a* 18S* rDNA HRV IV sequence 100% (680/680 bp) identical to that from *S. stercoralis* isolated from dogs in Switzerland (accession nos. MH932095-MH932097), and from a human patient in Myanmar (accession no. AB923888.1) and correspond to the haplotype A of HVR IV. This haplotype has been observed in parasites from both dogs and humans and was assumed to characterise the zoonotic population of *S. stercoralis* [13, 18].

The DNA sequences obtained in this study were deposited in GenBank® (accession no. OM429360, OM420254, OM420255, OM420256) after trimming the primer-binding regions.

## Discussion

In Austria, infections with *S.* *stercoralis* in dogs are considered to be rare, and the last report was published in 1985 [12]. However, several case reports have been published in the last decades across Europe, giving the impression that *S.* *stercoralis* infections may be emerging [4, 7–9, 13, 14, 22]. This emergence might be driven by the increase in the number of dogs being imported, which might change the pattern of endemic regions in the future, as has been observed for other diseases [32–34]. Likewise, in the case reports of this study, the infection might have been imported. Case 1 was imported from Slovakia, where the infection is known to be endemic; consequently, the parasite could have been introduced with the dog, rather than acquired in Austria [5]. In case 2, perusal of available information revealed that two dogs had been imported from Russia before the first clinical signs were observed in the breeding unit. These dogs may have been infected as both human and canine *S.* *stercoralis* infections have been reported in Russia [6, 10]. However, due to the late diagnosis of the infections, it was not possible to determine definitively whether these infections were imported or not. Prevalence studies are needed to evaluate the current situation and monitor the development of *S.* *stercoralis* distribution in Austria and other European countries.

Although genetic analysis detected haplotypes with zoonotic potential [13, 18], humans in contact with the infected dogs did not show clinical signs of infection. In case 1, the owners tested negative for *S.* *stercoralis* by means of flotation and the Baermann funnel technique*.* A serological examination could have provided additional information about a possible contact of the owners with the parasite; however it was not performed [35].

In both cases reported in this study, no coproscopical examination was performed prior to intestinal biopsy (case 1) or necropsy (case 2) analysis, even though the dogs did show clinical signs that might indicate an endoparasitosis. Coproscopy might have been neglected in these cases as a *Giardia* infection had been already detected by a non-coproscopical method, and continuous prophylactic anthelminthic treatment was administered. However, prophylactic treatment is not a reliable procedure to rule out parasitic diseases [27], as shown in the present report. Considering that *Giardia* are also commensals, the relevance of the positive result of the SNAP *Giardia* test may have been overestimated [36]. An early analysis by the Baermann funnel technique could have significantly reduced the duration of illness and thereby the stress and suffering of the dog and owner in case 1, and might have prevented the fatal course of disease in case 2. In suspicious cases or unspecific clinical signs, the Baermann funnel technique should be included in the parasitological examination, particularly because it is a cost-effective and simple method [16, 24, 25, 37]. Nevertheless, infections might still be missed in some cases with the Baermann funnel technique also; repeated faecal examinations or modification of the Baermann technique with charcoal pre-incubation would facilitate the detection of such infections [37]. Additional methods, such as Koga agar plate culture or PCR, might increase sensitivity [24, 25, 38, 39]. Serological tests are also available, but they are not yet validated for use in dogs, and cross-reactivity with other parasites such as ascarids might be possible [5, 27].

Case 1 showed severe clinical signs, in contrast to the two other dogs in the same household which were also infected. Similarly, in case 2, two of the puppies died, but two further puppies from the same litter showed no clinical signs. However, this is not surprising as the clinical presentation of *S.* *stercoralis* infection can vary greatly [8, 16, 22, 27]. In humans, immunosuppressive diseases lead to a higher risk for infection [1, 40]. More severe cases have also been observed in dogs, in which immunosuppression was induced by prolonged glucocorticoid treatment [7, 41, 42]. This is in agreement with the deterioration of clinical signs in case 1 after glucocorticoid treatment. Co-infection with CBoV might have played a role in the course of the disease in case 2 [43], but comparison to the other puppies of the litter was not possible, as their status of infection was unknown. Furthermore, because a pooled sample was examined it is not certain if both puppies were infected with CBoV.

Despite the use of anthelminthic treatment with compounds containing moxidectin prior to diagnosis in case 1 and in the adult dogs in case 2, clinical *S.* *stercoralis* infection did occur. Treatment with moxidectin in case 1 led to the improvement of clinical signs, but elimination of *S.* *stercoralis* from the faeces was only achieved after treatment with ivermectin. One of the dogs living in the same household, which was infected at the time of the initial examination, was, however, successfully treated with moxidectin. Controlled efficacy studies in dogs comparing these two agents would provide more reliable information. In humans, comparable efficacy of both components has been described [44, 45]. Other case reports in dogs have described the efficacy of ivermectin [7, 8, 13, 27], moxidectin [22] and fenbendazole [26] with variable reliability, especially after a single course of treatment, highlighting that case reports are not evidence-based proof of compound efficacy and that controlled experimental and clinical studies with a higher number of dogs are needed. This underlines further the need for coproscopical follow-up examinations in the case of strongyloidosis.

## Conclusion

Despite the infrequent diagnosis of *S.* *stercoralis* in Austria, veterinary practitioners should be aware of this potentially fatal helminth infection and the additional need for the Baermann technique for dogs with gastrointestinal clinical signs even though it is usually used to detect lungworm larvae. Without this previous examination, special caution should be exercised in the use of glucocorticoids. Treatment can be challenging, with only certain macrocyclic lactones showing a reliable efficacy, and follow-up examinations are essential. Prevalence studies would contribute towards an assessmentof the infection risk and distribution of *S.* *stercoralis*, and efficacy studies are needed to show the best treatment options.

## Data Availability

Additional data can be provided on request.

## Abbreviations

BID: Twice a day
BW: Body weight
CAV: Canine adenovirus
CBoV: Canine bocavirus
CDV: Canine distemper virus
CHV-1: Canine herpesvirus
*COI*: Mitochondrial cytochrome oxidase subunit I gene
CPiV: Canine parainfluenza virus
CPV-2: Canine parvovirus
CRCoV: Canine respiratory coronavirus
HVR: Hypervariable region
IBD: Inflammatory bowel disease
L1: First-stage larva
L3: Third-stage larva
*MDR-1*: Multidrug-resistance gene
p.o.: Orally
s.c.: Subcutaneously
SID: Once a day
rDNA: Ribosomal DNA
